# Primary Adenosquamous Cell Carcinoma of the Ileum in a Dog

**DOI:** 10.3390/vetsci7040155

**Published:** 2020-10-14

**Authors:** Masashi Yuki, Roka Shimada, Tetsuo Omachi

**Affiliations:** 1 Yuki Animal Hospital, 2-99 kiba-cho, Minato-ku, Nagoya, Aichi 455-0021, Japan; karokaroka0811@yahoo.co.jp; 2 Patho Labo, 9-400 Oomurokougen, Ito, Shizuoka 413-0235, Japan; tetsuo-omachi@patho-labo.com

**Keywords:** adenosquamous cell carcinoma, dog, ileum

## Abstract

A 9-year-old male, castrated Chihuahua was examined because of a 7-day history of intermittent vomiting. A mass in the small intestine was identified on abdominal radiography and ultrasonography. Laparotomy revealed a mass lesion originating in the ileum, and surgical resection was performed. The mass was histologically diagnosed as adenosquamous cell carcinoma. Chemotherapy with carboplatin was initiated, but the dog was suspected to have experienced recurrence 13 months after surgery and died 3 months later. To our knowledge, this is the first case report to describe the clinical course of adenosquamous cell carcinoma in the small intestine of a dog.

## 1. Introduction

Lymphoma is the most common type of intestinal tumor in dogs, followed by adenocarcinoma, leiomyosarcoma, and gastrointestinal stromal tumor [1]. Adenosquamous cell carcinoma (ASCC) is defined as a malignant tumor with glandular and squamous components and metastatic potential [2]. ASCC of the gastrointestinal tract is extremely rare in dogs, having been previously reported only in the esophagus and colorectal region [3,4]. ASCC of the small intestine is extremely uncommon in humans, with only nine cases having been reported to date [5]. No detailed reports exist regarding ASCC in the small intestine of dogs. This report describes the clinical course following surgical resection and chemotherapy in a case of canine ASCC of the ileum.

## 2. Case Presentation

In Japan, there is no ethics committee for private-practice veterinary hospitals. Nevertheless, this research was conducted according to the ethical guidelines of the Japan Veterinary Medical Association. The samples were collected after obtaining written consent from the dog’s owner.

A 9-year-old male, castrated Chihuahua was brought to our veterinary hospital with a 7-day history of intermittent vomiting. Physical examination revealed a body weight of 1.90 kg and a body condition score of 1 (on a scale of 1–5); the dog was extremely thin. The body temperature was 38.7 °C, heart rate was 150 beats/min, and respiratory rate was 48 breaths/min. His general appearance was quiet, alert, and responsive. Abdominal palpation revealed the presence of fecal impaction in the caudal abdomen.

A hematological analysis indicated leukocytosis (17,700/μL; reference interval (RI), 6000–17,000/μL) with neutrophilia (16,638 neutrophils/μL (RI, 3000–11,500/μL) including 354 band neutrophils). Biochemical analyses revealed abnormalities, including an increased concentration of C-reactive protein (CRP) (4.4 mg/dL; RI, 0–1.0 mg/dL) and decreased concentrations of albumin (2.0 g/dL; RI, 2.6–4.0 g/dL) and total calcium (8.7 mg/dL; RI, 9.3–12.1 mg/dL). The in-house heartworm antigen test based on immunochromatography showed a negative result. Urinalysis revealed a specific gravity of 1.048 and weak proteinuria, and the urine protein/creatinine ratio was normal (0.08; RI, <0.5). Thoracic and abdominal radiography revealed no thoracic abnormalities; however, the caudal abdomen was unclear due to fecal impaction. Abdominal ultrasonography revealed a mass (approximately 2.5 cm in diameter) with mixed echogenicity. The mass was suspected to have originated in the intestinal tract. Fine-needle biopsy was not performed due to the distribution of many blood vessels within the mass. No intraperitoneal lymphadenomegaly was observed. Based on these results, the intestinal mass was considered to be the underlying cause of the severe weight loss and intermittent vomiting. Famotidine (1 mg/kg, per os, q 24 h) (Gaster tablet; LTL Pharma, Tokyo, Japan) was administered until the scheduled surgery date.

**Figure 1:**
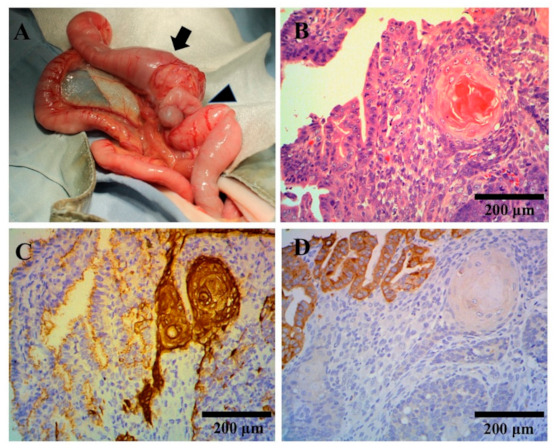
Macroscopic and histopathological tumor findings. (**A**) Macroscopic tumor findings. The mass originated from the ileum (arrow) proximal to the ileocecal (arrow head) region. (**B**–**D**) Histopathological tumor findings. Hematoxylin and eosin staining showed squamous and adenomatous components of the tumor (**B**). Immunohistochemical staining showed that cytokeratin (CK)5 and CK6 were more strongly stained in the squamous cell region (**C**) and CK8 was more strongly stained in the adenocarcinoma region (**D**).

On day 3, an additional blood test performed before surgery revealed normal total bile acid concentration (5.0 μmol/L; RI, 0–15.3 μmol/L), prothrombin time (7.4 s; RI, 7.4–8.4 s), and activated partial thromboplastin time (15.6 s; RI, 12.0–24.0 s) and a mild increase in fibrinogen concentration (376 mg/dL; RI, 150–350 mg/dL). Laparotomy was performed under anesthesia for resection of the mass. The mass originated from the ileum (3.5 cm × 3.0 cm × 2.5 cm). No intraperitoneal lymphadenomegaly and no peritoneal effusion were observed. Omental adhesions were observed in parts of the mass. The mass was macroscopically normal in color and appeared to have a thickened intestinal wall (Figure 1A). The portion of the ileum harboring the mass was resected, followed by end-to-end anastomosis. A white, encapsulated, nodular lesion with a smooth surface was found in part of the liver (2 mm × 2 mm × 2 mm); tissues from these liver lesions and mesenteric lymph nodes were sampled.

Postsurgical care included hospitalization and administration of lactated Ringer’s solution (5.0 mL/h, intravenous [IV]) (Lactec injection; Otsuka, Tokyo, Japan), meloxicam (0.2 mg/kg subcutaneous [SC], on first day) (Metacam; Boehringer Ingelheim, Tokyo, Japan), enrofloxacin (10 mg/kg, SC, q 24 h) (Baytril; Bayer, Tokyo, Japan), ampicillin (20.0 mg/kg, SC, q 12 h) (ampicillin; ZENOAQ, Fukushima, Japan), and famotidine (1 mg/kg, SC, q 24 h). On day 11, the dog was discharged following an improvement in vomiting condition compared with that on day 1.

Histopathological findings revealed that in the ileal mass, the mucosal epithelium exhibited neoplastic proliferation. Tumor cell atypia was moderately strong or moderate to moderately strong depending on the site, with a mitotic index of 75 (per 10 high-power fields). Tumor cells proliferated while forming lobular structures, showing a tendency of keratinization. Areas were also present in which tumor cells grew while forming irregular glandular cavities and lumens. The tumor comprised both malignant squamous and glandular components (Figure 1B). Tumor margins were negative at the proximal and distal ends; however, a small number of intravascular infiltrates were observed near the margin of the proximal end. Immunohistochemical staining of cytokeratin (CK) AE1/3, CK5, CK6 (anti-keratin/cytokeratin monoclonal antibody; NICHIREI, Tokyo, Japan), and CK8 (anti-keratin/cytokeratin monoclonal antibody; Progen Biotechnik GmbH, Heidelberg, Germany) revealed strong positive CK5 and CK6 staining in the squamous cell carcinoma (SCC) region, with weaker staining in the adenocarcinoma region (Figure 1C). CK8 was more strongly stained in the adenocarcinoma region, with weaker staining in the SCC region (Figure 1D). Both tumor cell types stained positive for CK AE1/3. Based on these findings, ASCC of the ileal mucosa was diagnosed. No tumor cells were found in the mesenteric lymph nodes or liver, and nodular lesions of the liver were hepatocyte hyperplasia. All pathological examinations and subsequent diagnoses were confirmed by board-certified veterinary pathologists at commercial laboratories (Patho Labo, Shizuoka, Japan).

By day 32, the dog’s weight had recovered to 2.1 kg, with good general condition. Albumin (2.6 g/dL) and CRP (<0.9 mg/dL) concentrations had also returned to within the RI. Thoracic and abdominal radiography and ultrasonography showed no abnormalities. On the same day, carboplatin chemotherapy (300 mg/m^2^, IV, every 3 weeks) (carboplatin intravenous infusion; Sandoz, Tokyo, Japan) was initiated based on histopathological findings of tumor vascular invasion. By day 123, the dog’s weight had recovered to 3.0 kg (body condition score of 3), and no abnormalities were observed in blood chemistry analysis or thoracic and abdominal radiography and ultrasonography. Five rounds of carboplatin chemotherapy were completed and discontinued on day 123.

**Figure 2:**
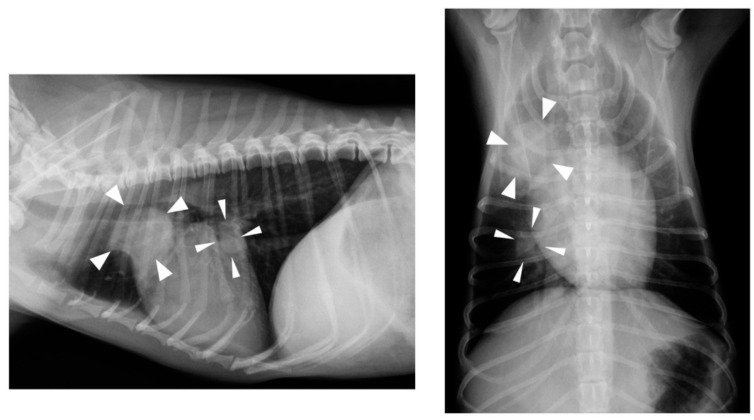
Thoracic radiography findings on day 404. Nodular lesions (arrow heads) were detected in the cranial and middle lobe of the right lung.

At a regular medical checkup on day 404, the dog weighed 3.1 kg and was in good general condition; however, an increase in CRP concentration (6.0 mg/dL) was observed. Thoracic radiography revealed nodular lesions in the right cranial lobe (approximately 2.0 cm in diameter) and the middle lobe of the right lung (approximately 1.0 cm in diameter) (Figure 2). Abdominal ultrasonography revealed hyperechoic nodular lesions in the right lobe of the liver (approximately 0.8 cm in diameter). Fine-needle aspiration was performed on liver nodules, but only a small number of hepatocytes were collected, and no significant findings were noted. These findings strongly suggested metastases of the ileal ASCC, but the owner opted against further examination and treatment. On day 511, the owner informed us that the dog had passed away; however, no specific details were provided.

## 3. Discussion

ASCC is defined as a carcinoma containing adenocarcinomatous and squamous cell carcinomatous components [6]. The histogenesis of ASCC has not yet been clearly elucidated, but the following four mechanisms have been hypothesized: (1) pluripotent epithelial stem cells capable of inducing malignant transformation of both cell types, (2) squamous metaplasia in the intestinal mucosa, (3) adenocarcinoma transforming into SCC, and (4) collision of both types of malignant tumors [7]. The third hypothesis is currently the most widely accepted: ASCC occurs through metaplastic malignant squamous transformation of adenocarcinomas [8].

In humans, reports of ASCC in the small intestine are extremely rare [5]. In four of the nine human cases reported thus far, metastasis had occurred by the time of initial diagnosis. Excluding those who received chemotherapy, all patients succumbed to the disease within 1 year of undergoing surgery [5]. ASCC reportedly shows more aggressive behavior and is associated with poorer prognosis than conventional adenocarcinomas [7]. In animal models, the SCC components of ASCC are known to grow more aggressively than adenocarcinoma components [9]. Moreover, several studies have reported that the doubling times for SCCs are significantly shorter than that for adenocarcinomas, suggesting that the SCC proportion in ASCC increases with tumor progression and may also be associated with prognosis [10]. In this study, the ileum was diagnosed as the primary site based on the lack of evidence of ASCC elsewhere. At the time of diagnosis, no evidence of metastases was found. These results were supported by good condition for more than a year after surgery. Unidentified nodular lesions in the lungs and liver could be lung or liver tumors that are completely different from ASCC. However, from the clinical course, recurrence of ASCC at approximately 400 days was strongly suspected, with death occurring at approximately 500 days. In dogs, the median survival rate after surgical treatment of adenocarcinoma in the small intestine is 7–10 months [11,12]. Although ASCC was not been formally identified as the cause of death in this case, survival was reasonably long compared with that for adenocarcinomas. There is limited information about the behavior of ASCC in dogs; therefore, this result is unclear. Based on human reports, this case of ASCC may have had a smaller SCC component. However, this case study was limited to the observation of a part of the pathological tissue, and the SCC proportion in the entire tumor was not investigated. In this case, surgery was considered effective for ASCC as it sufficiently improved the quality of life.

Considering that histopathological findings showed vascular invasion, chemotherapy was performed in this case. As there are no reports in the literature regarding chemotherapy for ASCC in dogs, carboplatin was administered with reference to chemotherapy for intestinal carcinoma [13]. There is currently no evidence to suggest that chemotherapy is the best treatment option owing to the small number of ASCC cases in humans. Therefore, there is also no established protocol, but chemotherapy may be administered in some cases with potentially poor prognosis. The effect of chemotherapy on ASCC in the present case is unknown, and it is necessary to accumulate cases to develop a better understanding.

In this study, the CRP concentration was elevated at the time of diagnosis of ASCC. CRP is an acute-phase protein produced in the liver in response to an increase in the release of pro-inflammatory cytokines such as interleukin-1, interleukin-6, and tumor necrosis factor during inflammation [14]. The CRP concentration is known to be elevated in many solid tumors [15,16]. It has also been reported that measuring CRP concentrations may be useful to monitor the response to treatment [16]. As the CRP concentration decreased rapidly after surgical removal of ASCC, CRP concentrations were measured in conjunction with imaging to monitor recurrence. As a result, an increased CRP concentration and an imaging finding suggestive of recurrence were obtained at the same time. Therefore, CRP measurement may be useful as a biomarker for the detection of ASCC recurrence.

To the best of our knowledge, this case report is the first to describe the clinical course of ASCC in the small intestine of a dog.
